# Universal health coverage and COVID-19: recent developments and implications

**DOI:** 10.1186/s40545-021-00306-x

**Published:** 2021-02-10

**Authors:** Rabia Hussain, Sara Arif

**Affiliations:** 1 Faculty of Pharmacy, The University of Lahore, Lahore, 54590 Pakistan; 2 Commonwealth Pharmacists Association, London, E1W 1AW UK; 3 Primary and Secondary Healthcare Department, Tehsil Headquarter Hospital Lahore Cantt, Lahore, Pakistan

**Keywords:** Universal health coverage, COVID-19, Healthcare, Medicines, Pakistan, Health system

## Abstract

Universal health coverage (UHC) is meant to access the key health services including disease prevention, treatment, rehabilitation, and health promotion. UHC varies according to demographics, epidemiology, and technology-based trends, as well as according to people’s expectations. Globally, the transition towards UHC has been associated with the intent of improving accessibility and affordability of healthcare. The COVID-19 pandemic has disrupted the health systems of even the most developed economies of the world in an unprecedented manner. The situation is also very challenging for the countries with the existing health inequities as well as the countries with the developing healthcare systems. This has amplified the need to accelerate efforts to build strong and resilient health systems to achieve progress towards UHC. This commentary discusses a global overview of UHC in the wake of COVID19. It also highlights the initiatives taken by Pakistan to promote the goals of UHC.

## Background

Universal health coverage (UHC) is defined by the World Health Organization as ensuring access to key health services including disease prevention, treatment, rehabilitation, and health promotion. Thus, promoting equity, efficiency effectiveness and affordability of healthcare services to the beneficiaries. UHC varies according to the demographics, epidemiology, and technology-based trends, as well as people’s expectations [1, 2]. It holds an equal importance in providing quality health services while ensuring equity and access together with financial management. Broader coverage of good quality health services results in improving the health indicators of the population, reduces health inequalities, and promotes stronger economic development. It also focuses on building resilient healthcare systems with the ability to address complex challenges through prevention, detection, timely response, maintenance of peace as well as to protect the economy [3].

## Global overview of universal health coverage adoption

Globally, the transition towards UHC has been associated with the intent of improving accessibility and affordability of healthcare, but UHC initiatives are often adopted in response to a social, economic, or political revamp. For example, Japan began its movement towards UHC before World War II to develop a healthy workforce through Citizens Health Insurance. France has provided UHC to all of its residents in 1999 by the establishment of the Universal Health Coverage Act (CMU), and it is the highest financial protection provider for healthcare related expenses among countries in the Organization for Economic Co-operation and Development (OECD) [4]. In some countries, UHC was adopted to counter financial crisis, such as Turkey, Indonesia, and Thailand. To overcome health inequalities and financial risk, Turkey has introduced UHC through the Health Transformation Programme in 2003 [4]. Similarly, Thailand adopted UHC in 2002, which provided health coverage to all 66.3 million Thai citizens; however, UHC in Indonesia was planned to be fully implement in 2019 through the National Health Insurance (NHI) enrolling about 198 million Indonesians in the programme. Similarly, after the establishment of the Unified Health System in 1988, more than 75% of the Brazilian nationals are now getting benefit from this programme. Likewise, South Africa is also progressing towards the implementation of UHC through the development of a National Health Insurance (NHI) programme [4, 5].

By the mid of twentieth century, high-income countries, such as New Zealand, Australia and Canada has implemented the UHC in various formats. For example, Australia and Canada have adopted UHC through the involvement of federal government and regionally administered systems in states. In 1938, after the implementation of Social Security Act, New Zealand had a consensus that the government will have a fundamental role in providing for the population’s healthcare needs [6].

## COVID-19 and universal health coverage

Amid COVID-19, the delivery of essential healthcare services is interrupted and inaccessible in almost all parts of the world. Even high-income countries have struggled hard to provide adequate healthcare, medical commodities, diagnostic testing, and specialized Intensive Care Unit (ICU) equipments during the pandemic. The situation is far more challenging for the countries with existing health inequities and developing healthcare systems. The pandemic has emphasized the importance of universal access to healthcare, proving that if the healthcare needs of a segment of the population are unattended or even a single individual is left unaddressed, the whole population is at risk [3].

Given the gross inequality in health status between male and female, rich and poor, developed and developing countries, it is estimated that about 150 million individuals worldwide face catastrophic healthcare expenditures and out of these more than 100 million are living in the poverty [7]. Thus, unlike any other crisis in the recent history, COVID-19 has amplified the urgency to accelerate efforts to build strong and resilient health systems and to achieve progress towards UHC. Strong health systems with adequate resources are key to a successful crisis response and management. It has been demonstrated that the countries with a strong UHC, such as South Korea and Singapore, have outperformed during COVID-19 pandemic. In Japan, a COVID-19 response is coupled with effective public financing policies and universal health insurance with uniform fee schedule. These policies have been effective in tackling COVID-19 and have provided an efficient mitigation response along with the restoration of essential health services amid pandemic [8].

## The emerging story of universal health coverage from Pakistan

Healthcare in Pakistan is facing enormous challenges involving a high burden of communicable and non-communicable diseases. Pakistan has highest prevalence of conditions including tuberculosis, maternal health issues, nutritional deficiencies, polio, malaria, and other communicable diseases in high-risk population groups [9–11]. Healthcare standards for access and quality lag far behind as compared to neighboring South Asian countries with Pakistan being ranked at 154th position among 195 countries worldwide. However, with an increase in Healthcare Access and Quality (HAQ) index from 26.8 to 37.6, access to healthcare has improved since the 1990s [12]. Despite the gains, geographical disparities still exist in the coverage of different healthcare services between provinces as well as between rural and urban areas [13]. Lower per capita spending on health, poor infrastructure, undue pressure and inadequate facilities at the public healthcare sector, lack of essential medicines and supplies, shortage of trained and skilled healthcare personnel, and high bed to patient ratios are some challenges that have worsened during pandemic. In nutshell, the COVID-19 crisis has highlighted the need to intensify efforts to improve UHC in Pakistan [3].

Pakistan is attempting to make progress in universal healthcare access with special attention being paid to vulnerable population groups. The Pakistan’s National Health Vision 2016–2025 has recognized the UHC as a top priority and several national programs and policies are aligned to it [10]. In this regard, several pro-poor initiatives have been taken by the government of Pakistan. “Sehat Sahulat Program”, which is a government-run health protection initiative, was started in 2016, providing financial coverage and accessibility to the households below the poverty line through micro health insurance scheme. There are now over seven million families currently enrolled in the programme, availing to the access of good quality medical care worth about 720,000 Pakistani rupees (PKR) (about 4600 USD) per family per year from both the public and private specialty based tertiary care and specialty hospitals. Similarly, on August 21, 2020. Khyber Pakhtunkhwa, a province in Pakistan, has launched a UHC programme for all its 40 million residents, as a response to COVID-19. This is a six-phased programme and it has completed on January 31, 2021, as being the first UHC programme in Pakistan to cover 100% of the population. It provides PKR200,000 (USD1200) for a range of services including—emergency and maternity care, orthopedic and general surgery; cardiac, neurological and kidney diseases, diabetes, breast cancer screening and artificial limbs (prosthesis), cancer treatment, kidney transplant, and ICU care [14]. These models are constructed in alignment with Pakistan’s commitment to achieve UHC by 2030. Insurance schemes under UHC can play a critical role by incorporating quality assurance into organizational procurement practices for a better access to healthcare [15]. The most significant initiative for Pakistan has been the development of National Package of Essential Health Services over the past 2 years based on Disease Control Priorities 3rd edition.

With this, Pakistan has become the first country to develop an evidence-based UHC services package that is focused on the equitable provision of essential healthcare services to each and every segment of the population through five service delivery platforms, i.e., the community level, primary healthcare, first level hospitals, tertiary hospitals and at population level. This package encompasses the sets of priority health interventions and intersectoral policies that are based on the localized evidence of burden of disease, feasibility, cost effectiveness, financial risk protection, equity, and other social indicators in Pakistan. Successful implementation of EPHS package through active involvement of all the stakeholders will gauge the progress towards achievement of UHC milestones within the available resources [16].

In Pakistan context, strengthening health and pharmacy systems and using generic medicines as cost-effective options can also help improve medicines use and can also help achieve the aims of UHC [17–19].

## Conclusions

UHC is meant to provide access to healthcare services to all parts of the population, regardless of geography, epidemiology and technology. In the past, many countries adopted UHC, particularly during economically difficult circumstances, so is the situation with the developing countries such as Pakistan during COVID-19. Although it will not be easy to fully achieve UHC in Pakistan, the government and respective partners need to work together to fully implement UHC in all the parts of the country.
